# The endoplasmic reticulum stress sensor IRE1α modulates macrophage metabolic function during *Brucella abortus* infection

**DOI:** 10.3389/fimmu.2022.1063221

**Published:** 2023-01-03

**Authors:** Erika S. Guimarães, Marco Túlio R. Gomes, Rodrigo C. O. Sanches, Kely Catarine Matteucci, Fábio V. Marinho, Sergio C. Oliveira

**Affiliations:** ^1^ Departamento de Genética, Ecologia e Evolução, Programa de Pós-Graduação em Genética, Instituto de Ciências Biológicas, Universidade Federal de Minas Gerais, Belo Horizonte, Minas Gerais, Brazil; ^2^ Departamento de Bioquímica e Imunologia, Instituto de Ciências Biológicas, Universidade Federal de Minas Gerais, Belo Horizonte, Minas Gerais, Brazil; ^3^ Departamento de Bioquímica e Imunologia, Faculdade de Medicina de Ribeirão Preto, Universidade de São Paulo, Ribeirão Preto, Brazil; ^4^ Plataforma de Medicina Translacional Fundação Oswaldo Cruz/Faculdade de Medicina de Ribeirão Preto, Universidade de São Paulo, Ribeirão Preto, Brazil; ^5^ Departamento de Imunologia, Instituto de Ciências Biomédicas, Universidade de São Paulo, São Paulo, Brazil

**Keywords:** UPR, endoplasmic reticulum stress, IRE1α, immunometabolism, HIF-1α, brucella abortus

## Abstract

Endoplasmic reticulum (ER) stress plays a major role in several inflammatory disorders. ER stress induces the unfolded protein response (UPR), a conserved response broadly associated with innate immunity and cell metabolic function in various scenarios. *Brucella abortus*, an intracellular pathogen, triggers the UPR *via* Stimulator of interferon genes (STING), an important regulator of macrophage metabolism during *B. abortus* infection. However, whether ER stress pathways underlie macrophage metabolic function during *B. abortus* infection remains to be elucidated. Here, we showed that the UPR sensor inositol-requiring enzyme 1α (IRE1α) is as an important component regulating macrophage immunometabolic function. In *B. abortus* infection, IRE1α supports the macrophage inflammatory profile, favoring M1-like macrophages. IRE1α drives the macrophage metabolic reprogramming in infected macrophages, contributing to the reduced oxidative phosphorylation and increased glycolysis. This metabolic reprogramming is probably associated with the IRE1α-dependent expression and stabilization of hypoxia-inducible factor-1 alpha (HIF-1α), an important molecule involved in cell metabolism that sustains the inflammatory profile in *B. abortus*-infected macrophages. Accordingly, we demonstrated that IRE1α favors the generation of mitochondrial reactive oxygen species (mROS) which has been described as an HIF-1α stabilizing factor. Furthermore, in infected macrophages, IRE1α drives the production of nitric oxide and the release of IL-1β. Collectively, these data unravel a key mechanism linking the UPR and the immunometabolic regulation of macrophages in *Brucella* infection and highlight IRE1α as a central pathway regulating macrophage metabolic function during infectious diseases.

## Introduction

The Endoplasmic reticulum (ER) is a central organelle responsible for synthesis, processing, and folding of secreted and transmembrane proteins. Physiologic stresses, such as increased secretory load, or pathological stresses, such as inflammatory challenges, cause an imbalance between protein load and the ER folding capacity, resulting in accumulation of misfolded proteins (1).Thenceforward, ER stress can induce the unfolded protein response (UPR), a physiological response aimed to restore ER homeostasis and preserve cellular functions (2, 3). The UPR consists of three major signaling pathways, activated by protein sensors: PKR-like ER kinase (PERK), activating transcription factor 6α (ATF6) and inositol-requiring enzyme 1α (IRE1α) (4). IRE1α, the most conserved UPR stress sensor, has been involved in a variety of cellular processes (5, 6), being considered a metabolic stress sensor and broadly associated with several metabolic disorders (6, 7).

*Brucella*, the etiologic agent of brucellosis, the most prevalent bacterial zoonosis worldwide (8), induces the UPR upon trafficking to the ER (9–11). Moreover, we and others have shown that *Brucella* infection activates the IRE1α axis of the UPR (9, 11, 12). Remarkably, full UPR induction during *B. abortus* infection in macrophages requires Stimulator of interferon genes (STING) (9). STING is an ER-transmembrane protein that was recently implicated in the metabolic reprogramming of *Brucella*-infected macrophages (13). Nevertheless, the possible interaction between the UPR and macrophage metabolic function in *Brucella* infection is poorly understood.

Macrophage metabolic reprogramming refers to the process whereby macrophages phenotypically mount a specific functional response to distinct microenvironment stimuli and signals. In this regard, macrophage polarization is not fixed (14), and two distinct populations, inflammatory M1 and anti-inflammatory M2 macrophages, represent the opposing ends of the full spectrum of macrophage polarization. Macrophages that display the M1 phenotype express various pro-inflammatory components such as nitric oxide (NO) and reactive oxygen species (ROS). By contrast, M2 phenotype macrophages are associated with generation of interleukin (IL)-10 and relate with tissue remodeling and wound healing (15). Regarding cellular metabolism, M1 macrophages energy production shifts from mitochondrial oxidative phosphorylation (OXPHOS) to glycolysis to support macrophage function (16). Our group recently demonstrated that the macrophage metabolic reprogramming during *B. abortus* infection is regulated by STING *via* HIF-1α (13), a global regulator of cellular metabolism that sustains the inflammatory phenotype in macrophages (17). Therefore, we aimed to determine the role of the UPR in mediating macrophage metabolic function in *B. abortus* infection. Here, we defined that IRE1α supports the inflammatory profile in macrophages and modulates its metabolic function. Notably, IRE1α is important for the metabolic shift from OXPHOS to an enhanced glycolytic metabolism that occurs upon *B. abortus* infection. Furthermore, IRE1α induces the generation of mitochondrial ROS (mROS) and HIF-1α stabilization. Additionally, IRE1α enhances canonical and non-canonical inflammasome activation, IL-1β release, NO production and cytokine secretion in infected macrophages, supporting the inflammatory profile in macrophages.

## Material and methods

### Mice

C57BL/6 animals were obtained from the Federal University of Minas Gerais (UFMG). HIF-1α conditional knockout mice in their myeloid cell lineage, termed here as HIF-1α KO (LysM-Cre^+/-^/HIF-1α^fl/fl^); HIF-1α-non-deletable littermate controls negative for Cre recombinase, termed here as HIF-1α WT (LysM-Cre^-/-^/HIF-1α^fl/fl^), were donated by Dr. Jose Carlos Alves-Filho (Ribeirão Preto Medical School, University of Sao Paulo, Brazil). Animals were maintained at UFMG and used at 6-8 weeks of age. All animal experiments were conducted in agreement with the Brazilian Federal Law number 11,794 and were preapproved by the Institutional Animal Care and Use Committee of the Federal University of Minas Gerais (CEUA no. 87/2017).

### Bacterial strain

*Brucella abortus* virulent strain S2308 was acquired from our laboratory collection. Prior to infection, *Brucella* was grown in *Brucella* broth medium (BD Pharmingen, San Diego, CA) under constant agitation for 3 days at 37°C.

### Bone marrow-derived macrophages

Bone marrow-derived macrophages (BMDMs) were generated as previously described, with some adaptations (18). Briefly, bone marrow cells from tibias and femurs were isolated and differentiated in DMEM (Gibco/Thermo Fisher Scientific, Waltham, MA) supplemented with 20% LCCM, 10% fetal bovine serum (FBS) (Life Technologies, Carlsbad, CA), 100 U/ml penicillin-streptomycin (Life Technologies), 1% HEPES (Life Technologies) at 37°C in 5% CO_2_ for 7 days until use. Then, unless otherwise specified, 5 x 10^5^ macrophages were seeded in 24 wells culture plates and cultivated in DMEM supplemented with 10% FBS, 100 U/ml penicillin-streptomycin and 1% HEPES at 37°C in 5% CO_2._

### UPR treatment and macrophage infection with *Brucella*

Macrophages were treated with 1 μg/mL of the ER stress inducer Tunicamycin (Sigma-Aldrich, St. Louis, MO) for 6 hrs (9). Where indicated, macrophages were pretreated with 50 μM 4μ8c (Sigma-Aldrich) for 30 min (9), with 1mM of 2-DG (Sigma-Aldrich) for 4 hrs (13) or with 0.5 mM Mito-TEMPO (Sigma-Aldrich), an mitochondrial superoxide scavenger, for 1 hr (19). Then, macrophages were infected *in vitro* with *B. abortus* in DMEM (5.5 mM glucose, 2 mM L-glutamine and no pyruvate) supplemented with 1% FBS for 24 h at 37°C in 5% CO_2_ at the multiplicity of infection (MOI) of 100:1, as previously described (13). Cellular lysates and culture supernatants were collected and stored at -80°C.

### Knockdown *via* small interfering RNA

BMDMs were transfected with small interfering RNA (siRNA) from siGENOME SMARTpools (Dharmacon, Lafayette, CO), according to the manufacturer’s instructions, using the GenMute siRNA transfection reagent (SignaGen Laboratories, Rockville, MD). Since IRE1α is ubiquitously expressed, the X-box binding protein 1 (XBP1) is a specific downstream target of the activation of the IRE1α axis of the UPR (20) used to asses IRE1α activation. Therefore, siGENOME SMARTpool siRNAs specific for mouse XBP1 (M-040825-00-0005) (siXBP1) and a control siRNA pool (D-001206-14-05) (siCNT) were used in this study. Forty-six hours after siRNA transfection, cells were infected with *B. abortus* as described above. Cellular lysates and culture supernatants were collected and stored at -80°C.

### qPCR analysis

Samples were resuspended in TRIzol (Invitrogen, Carlsbad, CA) to isolate total RNA in conformity with the manufacturer’s instructions. Genomic DNA was removed from total RNA by treatment with DNase I (Invitrogen). According to the manufacturer’s guidelines, reverse transcription of 1 μg of total RNA was performed using the Illustra Ready-To-Go RT-PCR Beads (GE Healthcare, Chicago, IL). Real-time RT-PCR was performed using SYBR Green PCR master mix (Applied Biosystems, Foster City, CA) on a QuantStudio3 real-time PCR instrument (Applied Biosystems), using the following parameters: 60°C for 10 min, 95°C for 10 min, 40 cycles of 95°C for 15 sec, and 60°C for 1 min, and a dissociation stage of 95°C for 15 sec, 60°C for 1 min, 95°C for 15 sec, and 60°C for 15 sec. The proper primers were used to amplify a specific fragment corresponding to specific gene targets as described: NOS2 forward: 5’- AGCACTTTGGGTGACCACCAGGA-3’, NOS2 reverse: 5’-AGCTAAGTATTAGAGCGGCGGCA-3’; IL-6 forward: 5’-CAGAATTGCCATCGTACAACTCTTTTC-3’, IL-6 reverse: 5’-AAGTGCATCATCGTTGTTCATACA-3’; TGF-β forward: 5’-CGCCATCTATGAGAAAACC-3’, TGF-β reverse: 5’-GTAACGCCAGGAATTGT-3’; YM1 forward: 5’-GGGCATACCTTTATCCTGAG-3’, YM1 reverse: 5’-CCACTGAAGTCATCCATGTC-3’; GLUT1 forward: 5’-GCTGTGCTTATGGGCTTCTC-3’, GLUT1 reverse: 5’-CACATACATGGGCACAAAGC-3’; HIF-1α forward: 5’-GGGTACAAGAAACCACCCAT-3’, HIF-1α reverse: 5’-GAGGCTGTGTCGACTGAGAA-3’, and β-actin forward: 5’- GGCTGTATTCCCCTCCATCG-3’, β-actin reverse: 5’-CCAGTTGGTAACAATGCCATGT-3’. The threshold cycle method was used to analyze all data. Data were analyzed as relative expression after normalization to the *β-actin* gene and fold changes are normalized to the non-infected. All measurements were conducted in triplicate.

### Cytokine measurements and nitric oxide assay

IL-6, IL-12, IL-1β, and TNF-α production in macrophages supernatants was assessed using ELISA (R&D systems, Minneapolis, MN), according to the manufacturer’s specifications. The NO assay was performed as previously described (21).

### Seahorse glycolytic rate analysis

The glycolytic profile of cells was assessed using the Extracellular Flux Analyzer XF96 (Agilent, Santa Clara, CA), as previously described (13). Briefly, 1 x 10^5^ macrophages were seeded per well on a Seahorse XF96 cell culture microplate and allowed to attach overnight. The next day, cells were treated or not with 50μM 4μ8c and infected or not with *B. abortus* for 24 hrs in culture medium. Proton efflux rate (PER), extracellular acidification rate (ECAR) and oxygen consumption rate (OCR) were determined using the Glycolytic Rate Assay Kit (Agilent) in accordance with the manufacturer’s instructions and as previously described (13). The Seahorse XF DMEM medium pH 7.4 (Agilent), supplemented with 4 mM L-glutamine, 2 mM pyruvate and 25 mM glucose was used during the assay. Experiments were executed in 5 replicates for each condition. MitoPER and glycoPER were calculated as previously described (13, 22).

### Measurement of mitochondrial reactive oxygen species

mROS were detected using MitoSOX Red (Invitrogen), a fluorescent dye specific for the detection of O_2_^-^ in the mitochondria. For confocal microscopy, MitoSOX Red staining evaluation was performed similarly as previously described (19). Briefly, 1 x 10^5^ macrophages were added on glass coverslips and non-infected or infected with *B. abortus* for 3 hrs or pre-treated with 50μM 4μ8c for 30 min and then infected with *B. abortus* for 3 hrs. Subsequently, cells were incubated with MitoSOX Red at a final concentration of 2.5 mM for 5 min, washed with Phosphate Buffered Saline (PBS) (Gibco), fixed with 4% formaldehyde, and washed once more with Phosphate Buffered Saline (PBS) (Gibco). Then, glass coverslips were mounted with Prolong Gold Antifade with DAPI (Invitrogen) and visualized by fluorescence microscopy, using equal settings. Three coverslips were analyzed per condition and representative images were taken using an ×40 objective using a Nikon A1 confocal microscope. The mean fluorescence intensity (MFI) for MitoSOX Red staining reflects mean fluorescence intensity x cell area and was quantified per whole cell. MFI was measured using ImageJ Software.

Generation of mROS was additionally evaluated by flow cytometry in macrophages as previously described (13). Briefly, macrophages were added to microcentrifuge tubes at a cell density of 5 x 10^5^. Then, cells were treated were indicated with 50μM 4μ8c for 30 min or with 0.5mM Mito-TEMPO for 1 hr and then infected or not with *B. abortus* for 1 hr. Then, cells were washed and resuspended in 200 μL PBS per microtube and transferred to a 96-well plate. mROS generation was assessed by flow cytometry using Attune Acoustic Focusing equipment (Life Technologies) and results were evaluated using FlowJo software. The data is expressed as MitoSOX Red median fluorescence intensity (MFI) fold change; particularly, mROS production by infected cells was relativized to mROS production by non-infected cell for each group, as previously described (13).

### Western blot analysis

Macrophages were lysed using the M-PER Mammalian Protein Extraction Reagent (Thermo Fisher Scientific) with 1:100 protease inhibitors (Sigma-Aldrich). Protein concentrations from macrophage lysates were determined by BCA assay and identical amounts of supernatants or lysates were loaded onto 12% or 15% SDS-polyacrylamide gel and transferred to nitrocellulose membranes (Amersham Biosciences) according to standard protocols. Membranes were treated for 1 hr in Tris-buffered saline (TBS) containing 0.1% Tween-20 containing 5% nonfat dry milk and incubated overnight with primary antibodies at 4°C, as previously described (13). Primary antibodies used are the following: a monoclonal antibody against IL-1β (clone 3A6, Cell Signaling Technology), a monoclonal antibody against HIF-1α (clone D1S7W, Cell Signaling Technology, Danvers, MA), a monoclonal antibody against caspase-11 (clone Flamy-1, Adipogen, San Diego, CA), a monoclonal antibody against gasdermin D (clone EPR19828, Abcam, Cambridge, U.K.), and a monoclonal antibody against the p20 subunit of caspase-1 (clone Casper-1, Adipogen), all at a 1:1000 dilution. A mouse monoclonal anti–β-actin (clone 13E5, Cell Signaling Technology) at a 1:5000 dilution was used as loading control. The blots were washed in TBS with 0.1% Tween 20 and incubated for 1 hr at 25°C with the suitable HRP-conjugated secondary antibody at a 1:1000 dilution. The bands were visualized in an Amersham Imager 600 (GE Healthcare) using Luminol chemiluminescent HRP substrate (Millipore).

### Lactate dehydrogenase release assay

The lactate dehydrogenase (LDH) enzyme activity was detected according to the manufacturer’s recommendations using a CytoTox96 LDH release kit (Promega, Madison, WI).

### Statistical analysis

Data analyses were performed using Student’s *t*- test, one-way ANOVA, or two-way ANOVA, as indicated, using GraphPad Prism 9 (GraphPad Software, San Diego, CA). A p value <0.05 (p<0.05) was considered statistically significant.

## Results

### The unfolded protein response supports the inflammatory profile in macrophages

**Figure 1:**
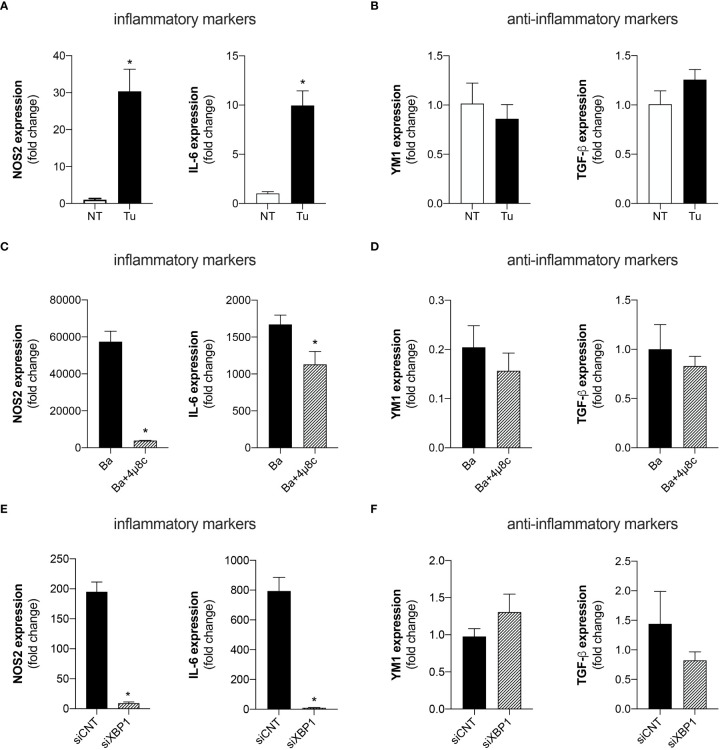
The UPR favors the polarization of inflammatory macrophages. **(A)** NOS2 and IL-6 expression levels determined by real-time PCR in macrophages from C57BL/6 mice non-treated (NT) or treated with Tunicamycin (Tu) (1 μg/mL). **(B)** YM1 and TGF-β expression levels determined by real-time PCR in macrophages from C57BL/6 mice non-treated (NT) or treated with Tunicamycin (Tu) (1 μg/mL). **(C)** NOS2 and IL-6 expression levels determined by real-time PCR in macrophages from C57BL/6 mice infected with *B abortus* (Ba) or pre-treated with 4μ8c (50 μM) and infected with *B abortus* (Ba+4μ8c). **(D)** YM1 and TGF-β expression levels determined by real-time PCR in macrophages from C57BL/6 mice infected with *B abortus* (Ba) or pre-treated with 4μ8c (50 μM) and infected with *B abortus* (Ba+4μ8c). **(E)** NOS2 and IL-6 expression levels determined by real-time PCR in macrophages from C57BL/6 mice subjected to specific XBP1 gene knockdown and then infected with *B abortus*. **(F)** YM1 and TGF-β expression levels determined by real-time PCR in macrophages from C57BL/6 mice subjected to specific XBP1 gene knockdown and then infected with *B abortus*. The data are representative of three independent experiments. The data are presented as mean ± SD, * p < 0.05, Student’s t test.

UPR signaling has been extensively associated with inflammatory responses in various settings (23, 24). Therefore, we investigated whether UPR regulates macrophage polarization i.e., the macrophage profile typically associated with an inflammatory (M1) or anti-inflammatory (M2) profile. Quantitative real-time RT-PCR analysis demonstrated that treatment of macrophages with Tunicamycin, a potent ER stress inducer, enhanced the expression of the inflammatory macrophage-related markers, NOS2 (inducible nitric oxide synthase) and IL-6 (**Figure 1A**). Remarkably, treatment with Tunicamycin did not alter the expression of anti-inflammatory markers such as, YM1 (chitinase-like 3) and TGF-β (transforming growth factor beta) compared to non-treated macrophages (**Figure 1B**). These results indicate that Tunicamycin-induced UPR favors macrophage polarization towards an inflammatory profile.

Considering that the UPR has been extensively described as crucial for inducing an appropriate inflammatory response during *Brucella* infection (9, 10, 25), we investigated whether the IRE1α axis of the UPR contributes to macrophage polarization. Inhibition of IRE1α by pre-treatment with 4μ8c (4-methyl umbelliferone 8-carbaldehyde), a potent and selective IRE1α inhibitor (26), reduced the expression of the inflammatory macrophage-related markers, NOS2 and IL-6 in *B. abortus-*infected macrophages (**Figure 1C**). Meanwhile, IRE1α inhibition did not alter the expression of the anti-inflammatory macrophage-related markers, YM1 and TGF-β in infected macrophages (**Figure 1D**).

Corroborating these results, knockdown of XBP1 [a specific downstream target of the activation of the IRE1α axis of the UPR (20)], *via* small interfering RNA, also reduced the expression of the inflammatory macrophage-related markers, NOS2 and IL-6, in *B. abortus-*infected macrophages compared to the control infected with *B. abortus* (**Figure 1E**), and did not alter the expression of the anti-inflammatory macrophage-related markers, YM1 and TGF-β, in infected macrophages (**Figure 1F**). Altogether, these results demonstrate that IRE1α modulates macrophage polarization, favoring the induction of inflammatory macrophages during *B. abortus* infection.

### The unfolded protein response modulates the inflammatory response in macrophages

**Figure 2:**
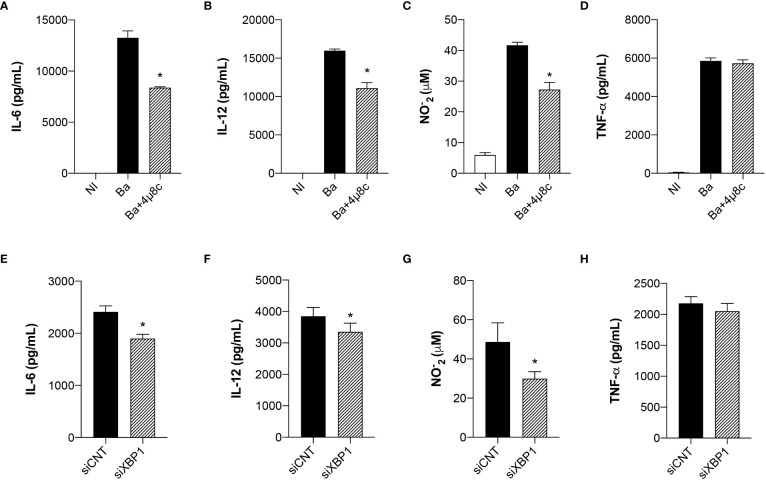
The UPR modulates the inflammatory response in infected macrophages. **(A)** IL-6, **(B)** IL-12 and **(D)** TNF-α produced by macrophages from C57BL/6 mice non-infected (NI), infected with *B abortus* (Ba) or pre-treated with 4μ8c (50 μM) and infected with *B abortus* (Ba+4μ8c), detected in cell supernatants using ELISA. **(C)** NO_2_^−^ (nitrite) accumulation in the media of macrophages from C57BL/6 mice non-infected (NI), infected with *B abortus* (Ba) or pre-treated with 4μ8c (50 μM) and infected with *B abortus* (Ba+4μ8c), measured by Griess reaction. **(E)** IL-6, **(F)** IL-12 and **(H)** TNF-α produced by macrophages from C57BL/6 mice subjected to specific XBP1 gene knockdown and then infected with *B abortus*. **(G)** NO_2_^−^ (nitrite) accumulation in the media of macrophages from C57BL/6 mice subjected to specific XBP1 gene knockdown and then infected with *B abortus*. The data are representative of three independent experiments. The data **(A–D)** are presented as mean ± SD, * p < 0.05, one-way ANOVA. The data **(E–H)** are presented as mean ± SD, * p < 0.05, Student’s t test.

ER stress is strongly associated with the immune signaling response to invading microorganisms (20). Given that the UPR regulates macrophage polarization during *B. abortus* infection, we evaluated cytokine secretion in *Brucella*-infected macrophages pre-treated with 4μ8c or transfected with small interfering RNA. Inhibition of IRE1α impaired IL-6 (**Figure 2A**) and IL-12 (**Figure 2B**) secretion and NO production (**Figure 2C**), inflammatory mediators typically associated with inflammatory macrophages (14), whereas TNF-α secretion was unaltered (**Figure 2D**). Furthermore, XBP1 silencing likewise reduced IL-6 (**Figure 2E**) and IL-12 (**Figure 2F**) secretion and NO production (**Figure 2G**), whereas TNF-α secretion was unaltered (**Figure 2H**). These results indicate that IRE1α has an important role in inducing pro-inflammatory responses during *B. abortus* infection.

### The metabolic reprogramming in infected macrophages is IRE1α-dependent

**Figure 3:**
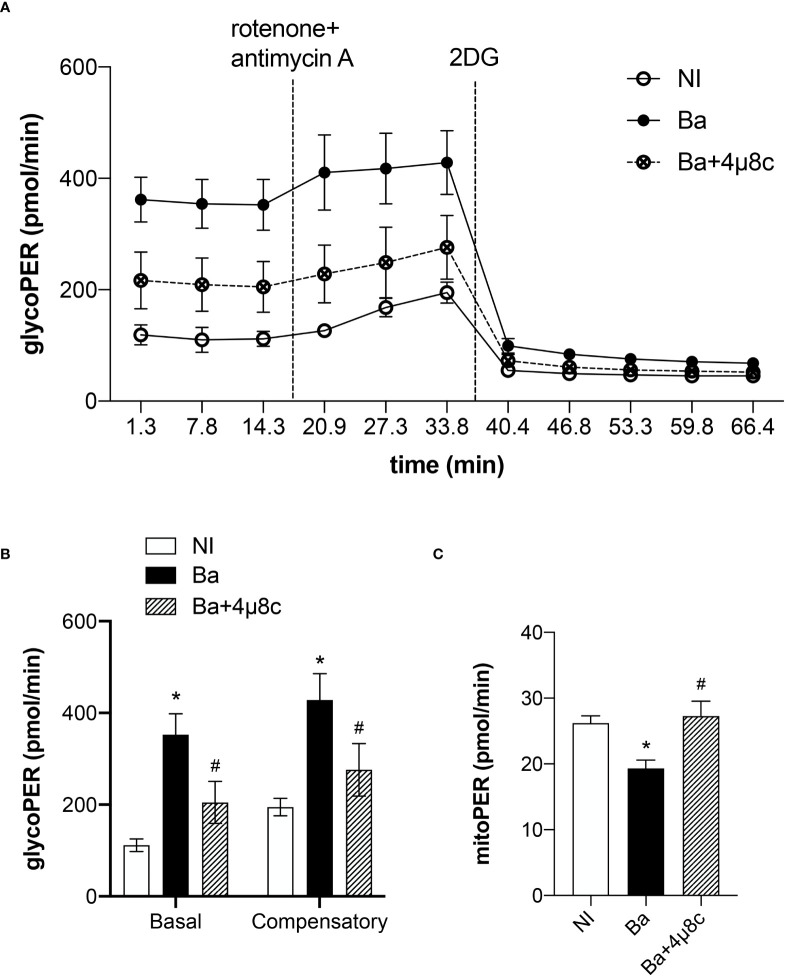
IRE1α regulates the metabolic function of infected macrophages. **(A)** Time-course quantification of glycolytic proton efflux rate (glycoPER) in macrophages from C57BL/6 mice non-infected (NI), infected with *B abortus* (Ba) or pre-treated with 4μ8c (50 μM) and infected with (*B abortus* (Ba+4μ8c). **(B)** Quantification of basal and compensatory glycoPER. **(C)** Quantification of mitoPER. The data are representative of three independent experiments. The data **(B, C)** are presented as mean ± SD, * p < 0.05, one-way ANOVA. * p < 0.05, compared to NI and # p < 0.05, compared to Ba, one-way ANOVA.

Macrophage polarization is closely associated with the metabolic rewiring required to sustain macrophage biological functions (16). Moreover, IRE1α senses cellular metabolic stressful conditions acting to sustain metabolic homeostasis (7). Therefore, we evaluated the role of the IRE1α in the metabolic function of macrophages in *Brucella* infection using a glycolytic rate assay. In that context, the glycolytic acidification was determined by calculating the glycolytic proton efflux rate (glycoPER) as previously described (13). The time-course measurements of glycoPER showed that macrophages infected with *B. abortus* displayed a higher proton flux rate due to the glycolytic acidification compared to non-infected macrophages (**Figure 3A**). The reduction of proton flux rate achieved after addition of the inhibitor 2-deoxy-D-glucose (2-DG) confirms that this acidification is provided by the glycolytic pathway (**Figure 3A**). In addition, we calculated the basal glycolysis levels, which were determined before OXPHOS blockage by rotenone and antimycin A, and the compensatory glycolysis levels, that were observed after OXPHOS inhibition. We demonstrated that *B. abortus* infection increased basal and compensatory glycolysis. Markedly, our results revealed that inhibition of IRE1α reduced the basal and compensatory glycolysis when compared to non-treated infected macrophages (**Figure 3B**). Moreover, we evaluated the acidification rate derived from the CO_2_ produced entirely by the mitochondria (mitoPER) as previously described (13) to evaluate OXPHOS. Remarkably, our results indicate that the decrease in OXPHOS induced by *B. abortus* occurs in an IRE1α-dependent manner as IRE1α inhibition restored OXPHOS to levels similar to non-infected cells (**Figure 3C**). Together these results indicate that IRE1α participates in the metabolic reprogramming of macrophages and is crucial for the increase in glycolysis and the reduction in OXPHOS observed during *Brucella* infection, distinctive metabolic features of inflammatory macrophages (16).

### IRE1α drives mROS generation in infected macrophages

The inflammatory profile in macrophages is characterized by the efficient generation of ROS (16). Furthermore, the cellular metabolic shift (from producing ATP mainly by OXPHOS to the increased production of ATP by glycolysis) repurposes mitochondria from ATP production to ROS generation, which promotes the inflammatory profile in macrophages (27). Therefore, we evaluated mROS production, to unravel how the UPR regulates the macrophage metabolic function during *B. abortus* infection.

**Figure 4:**
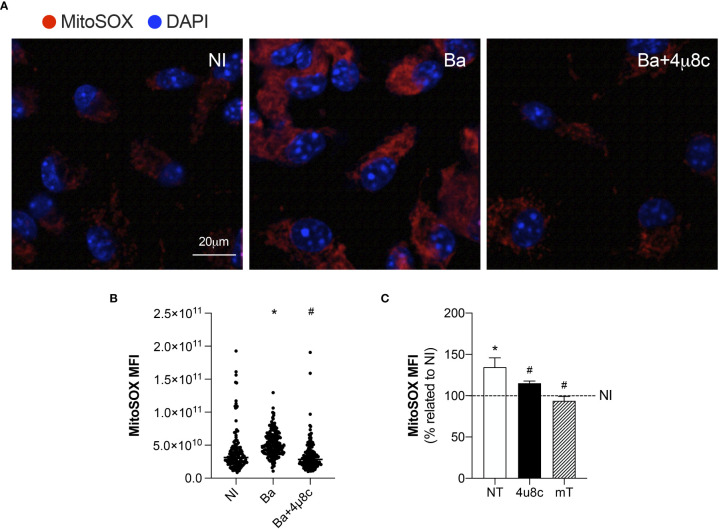
IRE1α drives mROS generation. **(A)** Representative confocal microscopy of MitoSOX Red staining in macrophages from C57BL/6 mice non-infected (NI), infected with *B abortus* (Ba) or pre-treated with 4μ8c (50 μM) and infected with *B abortus* (Ba+4μ8c). MitoSOX Red is in red, and nuclei (DAPI) is in blue. Scale bar, 20 μm. **(B)** MitoSOX Red mean fluorescent intensity, determined as described in Materials and Methods. **(C)** MitoSOX Red flow cytometry analysis of mROS production fold change induced by *B abortus* relativized to non-infected (NI) cells for each experimental group: non-treated (NT), pre-treated with 50 μM 4μ8c (Ba+4μ8c) or pre-treated with 0.5 mM Mito-TEMPO (mT). The data are representative of three independent experiments. The data **(B)** is presented as mean ± SD, * p < 0.05 (compared to NI) and # p < 0,05 (compared to Ba), one-way ANOVA. The data **(C)** is presented as mean ± SD, * p < 0.05 [compared to non-infected (NI, set to 100%)] and # p < 0.05 (compared to NT), one-way ANOVA.

Confocal microscopy analysis of MitoSOX Red, a mitochondrial superoxide indicator, demonstrated that infection with *B. abortus* enhanced mROS generation when compared to non-infected macrophages (**Figures 4A, B**). Moreover, inhibition of IRE1α reduced mROS production in infected macrophages to levels similar to those of non-infected (**Figures 4A, B**). Corroborating these results, MitoSOX Red flow cytometry analysis confirmed that infection with *B. abortus* enhanced mROS generation compared to non-infected macrophages, while IRE1α inhibition reduced mROS production compared to infected non-treated cells (**Figure 4C**). As expected, in infected macrophages, pretreatment with a scavenger specific for mROS, Mito-TEMPO, abrogated mROS production (**Figure 4C**). Altogether, these results strongly indicate that IRE1α contributes to mROS generation in *Brucella*-infected macrophages.

### IRE1α modulates HIF-1α stabilization in *Brucella*-infected macrophages

**Figure 5:**
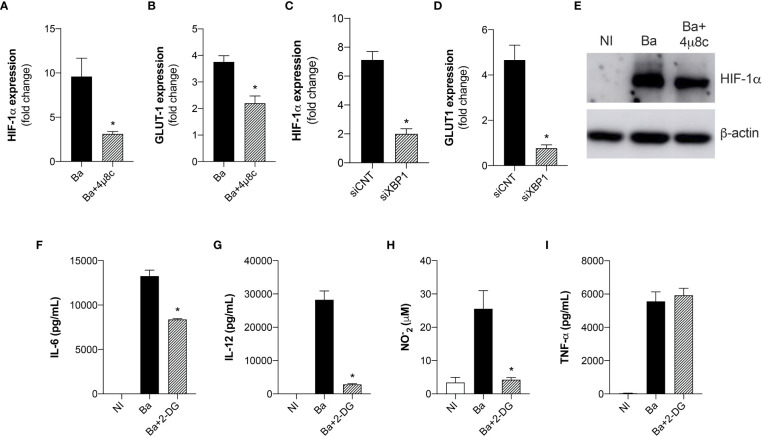
The expression and stabilization of HIF-1α in *Brucella*-infected macrophages requires IRE1α. **(A)** HIF-1α and **(B)** GLUT-1 expression levels determined by real-time RT-PCR in macrophages from C57BL/6 mice infected with *B abortus* (Ba) or pre-treated with 4μ8c (50 μM) and infected with *B abortus* (Ba+4μ8c). **(C)** HIF-1α and **(D)** GLUT-1 expression levels determined by real-time RT-PCR in macrophages from C57BL/6 mice subjected to specific XBP1 gene knockdown and then infected with *B abortus.* **(E)** Western blot analysis of HIF-1α in cell lysates from macrophages from C57BL/6 mice non-infected (NI), infected with *B abortus* (Ba) or pre-treated with 4μ8c (50 μM) and infected with *B abortus* (Ba+4μ8c). Equal loading was controlled by measuring β- actin in the corresponding cell lysates. **(F)** IL-6, **(G)** IL-12, **(I)** TNF-α produced by macrophages from C57BL/6 mice non-infected (NI), infected with *B abortus* (Ba) or pre-treated with 2-DG (1mM) and infected with *B abortus* (Ba+2-DG), detected in cell supernatants using ELISA. **(H)** NO_2_^−^ (nitrite) accumulation in the media of macrophages from C57BL/6 mice non-infected (NI), infected with *B abortus* (Ba) or pre-treated with 2-DG (1mM) and infected with *B abortus* (Ba+2-DG), measured by Griess reaction. The data are representative of three independent experiments. The data **(A–D)** are presented as mean ± SD, * p < 0.05, student’s t test. The data **(F–I)** are presented as mean ± SD, * p < 0.05, one-way ANOVA.

Recent reports have shown that mROS modulates HIF-1α activity (27). Moreover, it was recently demonstrated that mROS stabilizes HIF-1α expression in macrophages infected with *B. abortus* (13). Considering that HIF-1α drives the metabolic reprogramming in macrophages in *Brucella* infection (13), we addressed whether IRE1α could modulate HIF-1α expression and stabilization. Quantitative real-time RT-PCR analysis revealed that inhibition of IRE1α reduced HIF-1α (**Figure 5A**) and GLUT1 (glucose transporter 1, a marker for the HIF-1α-induced glycolysis) (**Figure 5B**) (28) expression compared to non-treated infected macrophages. Accordingly, XBP1 silencing also reduced HIF-1α (**Figure 5C**) and GLUT1 (**Figure 5D**) expression in infected macrophages compared to the infected control. Furthermore, inhibition of IRE1α reduced HIF-1α protein level in infected macrophages compared to non-treated infected macrophages (**Figure 5E**). Altogether, these results indicate that IRE1α contributes to HIF-1α expression and stabilization in *Brucella*-infected cells. Recent studies showed that HIF-1α and HIF-1α-induced glycolysis are important modulators of innate immunity (29, 30). Considering that the UPR is crucial for triggering immune responses against infections (20), we evaluated the role of glycolysis in the immune responses in *B. abortus-*infected macrophages. Inhibition of glucose flux using 2-DG in infected macrophages reduced IL-6 (**Figure 5F**), IL-12 (**Figure 5G**), and NO secretion (**Figures 5H**) compared to non-treated infected macrophages whereas TNF-α secretion was unaltered (**Figure 5I**). Overall, these results demonstrate that IRE1α modulates HIF-1α function and confirms that the HIF-1α-glycolysis axis contributes for triggering inflammatory responses in *B. abortus* infection.

### IRE1α-dependent stabilization of HIF-1α is required for induction of inflammatory responses in Brucella-infected macrophages

**Figure 6:**
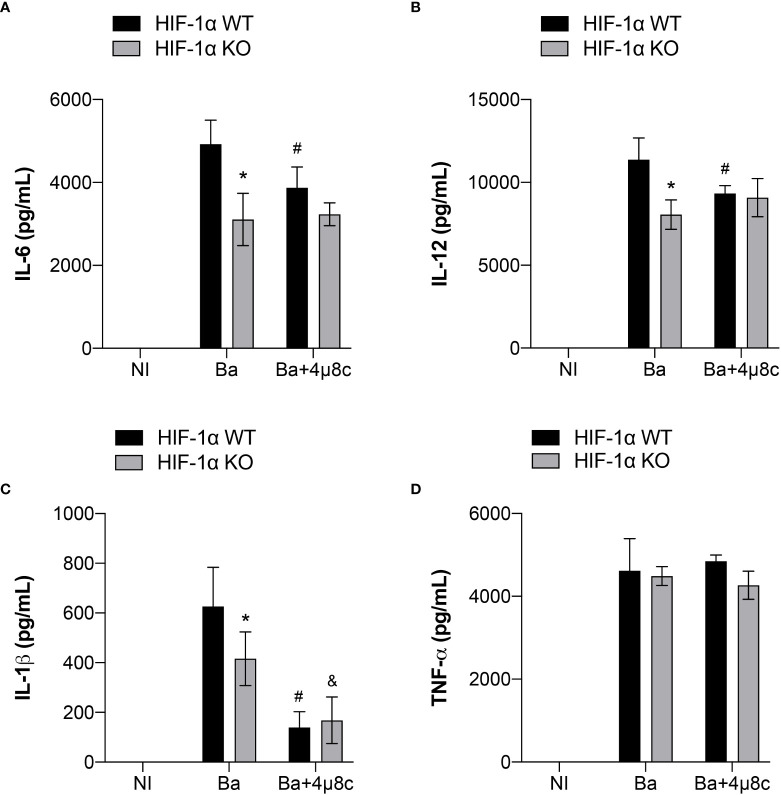
IRE1α is necessary for full induction of HIF-1α-dependent inflammatory responses during infection. **(A)** IL-6, **(B)** IL-12, **(C)** IL-1β and **(D)** TNF-α produced by macrophages derived from HIF-1α WT and HIF-1α KO mice non-infected (NI), infected with *B abortus* (Ba) or pre-treated with 4μ8c (50 μM) and infected with *B abortus* (Ba+4μ8c), detected in cell supernatants using ELISA. The data are representative of three independent experiments. The data are presented as mean ± SD, * p < 0.05 (compared between WT and KO), # p < 0.05 (compared to Ba from WT), & p < 0.05 (compared to Ba from KO), two-way ANOVA.

It is widely known that HIF-1α is involved in inflammation (31). Therefore, to further investigate the contribution of the IRE1α-HIF-1α axis in inducing inflammatory responses during *B. abortus* infection, we inhibited IRE1α in HIF-1α WT and HIF-1α WT KO in non-infected and infected macrophages and assessed the production of inflammatory cytokines. Lack of HIF-1α reduced IL-6 (**Figure 6A**) and IL-12 secretion (**Figures 6B**) in infected macrophages. Furthermore, absence of HIF-1α also impaired IL-1β release (**Figure 6C**) whereas TNF-α secretion was unaltered (**Figure 6D**).

In addition, inhibition of IRE1α using 4μ8c in HIF-1α WT infected macrophages reduced IL-6 (**Figure 6A**), IL-12 (**Figure 6B**) and IL-1β (**Figure 6C**) release compared to non-treated HIF-1α WT infected macrophages, whereas TNF-α secretion was unaffected (**Figure 6D**). Remarkably, inhibition of IRE1α in HIF-1α KO infected macrophages did not alter IL-6 (**Figure 6A**) and IL-12 (**Figure 6B**) secretion compared to non-treated HIF-1α KO infected macrophages. These data indicate that IRE1α induces the production of these cytokines, at least partially, in a HIF-1α-dependent fashion. Interestingly, inhibition of IRE1α further reduced IL-1β secretion (**Figure 6C**) in HIF-1α KO infected macrophages in comparison with non-treated HIF-1α KO infected macrophages. Collectively, these results suggest that the IRE1α-dependent stabilization of HIF-1α is indispensable for inducing the inflammatory responses against *B. abortus* infection.

### IRE1α induces inflammasome activation in *Brucella*-infected macrophages

**Figure 7:**
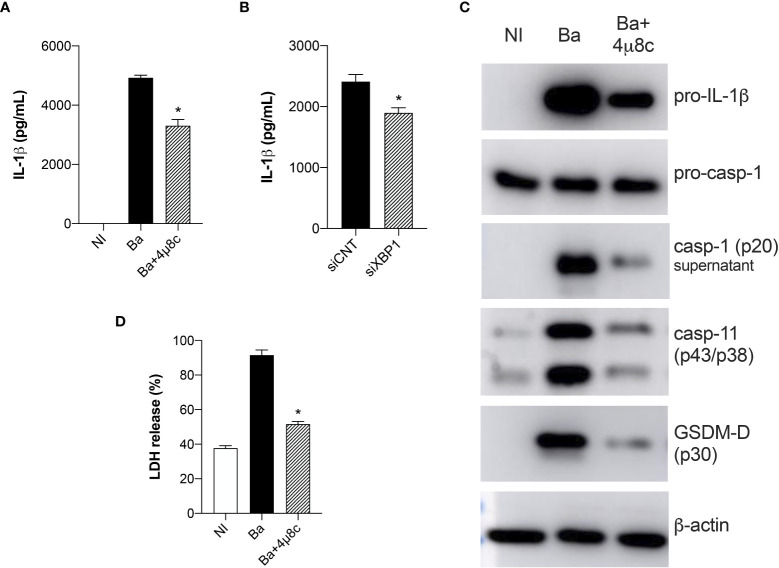
IRE1α induces inflammasome activation and IL-1β release in infected macrophages. **(A)** IL-1β released by macrophages derived from C57BL/6 mice non-infected (NI), infected with *B abortus* (Ba) or pre-treated with 4μ8c (50 μM) and infected with *B abortus* (Ba+4μ8c), detected in cell supernatants using ELISA. **(B)** IL-1β released by macrophages derived from C57BL/6 mice subjected to specific XBP1 gene knockdown and then infected with *B abortus*. **(C)** Western blot analysis of pro-IL-1β, pro-caspase-1, caspase-11 and p30 fragment of GSDMD in cell lysates and caspase-1 (p20 subunit) in supernatants from macrophages derived from C57BL/6 mice, non-infected (NI), infected with *B abortus* (Ba) or pre-treated with 4μ8c (50 μM) and infected with *B abortus* (Ba+4μ8c). Equal loading was controlled by measuring β-actin in the corresponding cell lysates. **(D)** LDH release in macrophages derived from C57BL/6 mice non-infected (NI), infected with *B abortus* (Ba) or pre-treated with 4μ8c (50 μM) and infected with *B abortus* (Ba+4μ8c). Values represent the percentage of LDH release compared with lysed cells. The data are representative of three independent experiments. The data **(A, D)** are presented as mean ± SD, * p < 0.05, one-way ANOVA. The data **(B)** are presented as mean ± SD, * p < 0.05, Student’s t test.

Here, we demonstrated that IRE1α is crucial for inducing IL-1β release both in HIF-1α WT and HIF-1α KO infected macrophages, suggesting an important role of IRE1α in regulating IL-1β release in *Brucella* infection. Therefore, to better address this, we evaluated inflammasome activation. Corroborating our data showing that inhibition of IRE1α reduced IL-1β secretion in HIF-1α WT and HIF-1α KO infected macrophages, inhibition of IRE1α also reduced IL-1β release in C57BL/6 wild type infected macrophages compared to non-treated infected cells (**Figure 7A**). Corroborating these data, XBP1 silencing also reduced IL-1β secretion in infected macrophages compared to the infected siRNA control (**Figure 7B**).

Furthermore, IRE1α inhibition reduced pro-IL-1β protein level in infected macrophages and also processing of caspase-1 (p20 subunit in supernatant) was reduced in 4μ8c-treated infected macrophages compared to non-treated infected cells (**Figure 7C**). Altogether these results indicate decreased canonical-inflammasome assembly upon IRE1α inhibition in infected macrophages.

In addition to canonical-inflammasome activation, *B. abortus* also activates the non-canonical inflammasome and IL-1β release in this case is partially dependent on caspase-11 and gasdermin-D (GSDMD) (32). Furthermore, activation of non-canonical inflammasome triggers pyroptosis and consequent membrane disruption, releasing lactate dehydrogenase (LDH) as well as other cytosolic contents (32). Hence, we further evaluated the role of IRE1α in non-canonical inflammasome activation. Inhibition of IRE1α reduced the intracellular protein level of caspase-11 and GSDMD cleavage (p30 fragment) in 4μ8c-treated infected macrophages compared to non-treated infected macrophages (**Figure 7C**). Regarding LDH, inhibition of IRE1α diminished LDH release in infected macrophages to levels similar to non-infected macrophages (**Figure 7D**), suggesting that IRE1α participates in non-canonical inflammasome activation and pyroptosis. Altogether, these results indicate that IRE1α is indispensable for canonical and non-canonical inflammasome activation and IL-1β release in *B. abortus*-infected macrophages.

## Discussion

The UPR has been linked to macrophage polarization in various settings and especially in metabolic disorders. For example, IRE1α specific deletion in adipose tissue promotes M2 and decreases M1 polarization of macrophages. This M1-M2 imbalance limits energy expenditure capacity and promotes insulin resistance in mice (33). Furthermore, IRE1α knockdown reduces M1 proinflammatory macrophages and promotes the M2-pheynotypic shift in macrophages in a mouse model of steatosis, aggravating the ischemia reperfusion injury of fatty liver (34). Accordingly, we demonstrated here that IRE1α inhibition reduces M1 macrophage polarization in *Brucella*-infected macrophages without affecting M2 macrophages, corroborating the UPR role as an important inflammatory signal during bacterial infections.

Regarding *B. abortus* infection, infected human-like and mouse macrophages undergo reprogramming that resembles the inflammatory macrophage profile also known as the Warburg effect (13, 35). We demonstrated that IRE1α modulates the macrophage metabolic function, supporting the metabolic shift towards glycolysis, unraveling the UPR as a regulator of macrophage metabolism during *Brucella* infection. A recent report from our group demonstrated that HIF-1α drives the metabolic reprogramming in infected macrophages (13). Accordingly, we demonstrated that IRE1α favors HIF-1α expression and stabilization, indicating that the IRE1α-dependent HIF-1α pathway may play a role in regulating the macrophage metabolic function during *B. abortus* infection. Previous reports suggest an intimate relationship between hypoxia responses and ER stress leading to macrophage polarization (36). For instance, the UPR enhances HIF-1α phosphorylation and interacts with hypoxia response pathways to augment HIF-1α mRNA expression (37). Moreover, the ubiquitin ligase Siah2 is another example of the interaction of HIF-1α and the UPR during hypoxia as it limits prolyl hydroxylase domains (PHD) protein availability during hypoxia, stabilizing HIF-1α (38). Notably, our results show a connection between HIF-1α and UPR that occurs outside of a hypoxic setting.

Regarding inflammasome activation, we demonstrated that IRE1α participates in IL-1β secretion and directly interferes with pro-IL-β synthesis. Additionally, IRE1α stimulates caspase-1 and caspase-11 activation and GSDMD cleavage, indicating its crucial function in inducing canonical and non-canonical inflammasome activation in *B. abortus* infection. Previous studies corroborate the involvement of the IRE1α in the activation of the NLRP3 canonical inflammasome. For example, IRE1α mediates saturated fatty-acid-induced activation of the NLRP3 inflammasome (39). Moreover, during irremediable ER stress, IRE1α promotes inflammasome activation by inducing Thioredoxin-Interacting Protein (TXNIP), which is crucial for pro-caspase-1 cleavage and IL-β secretion (40). Furthermore, it was previously demonstrated in macrophages that *Brucella*-induced mROS is crucial for NLRP3-caspase-1 inflammasome activation (19) and a previous report indicated that infection with an attenuated *Brucella* strain induces IRE1α and activates the inflammasome *via* NLRP3-driven mitochondrial damage (41). Accordingly, we demonstrated here that IRE1α drives the production of mROS in *Brucella*-infected macrophages.

Moreover, mROS stabilizes HIF-1α that induces canonical inflammasome activation in *B. abortus*-infected macrophages (13). Therefore, our results imply that IRE1α-dependent mROS might contribute to caspase-1 activation and inflammasome assembly *via* modulation of HIF-1α stabilization. Remarkably, HIF-1α is not required for non-canonical inflammasome activation in *B. abortus* infection (13), raising the hypothesis that the UPR may affect non-canonical inflammasome assembly in a HIF-1α-independent manner. This hypothesis is supported by our data concerning IL-1β release in HIF-1α KO macrophages. Whereas secretion of other inflammatory cytokines such as IL-6 and IL-12 relies on IRE1α-dependent induction of HIF-1α, IL-1β release in HIF-1α KO macrophages is further reduced upon treatment with 4u8c.

Intracellular bacteria uses host cell metabolites to survive and replicate (42). In that context, the macrophage metabolic profile is particularly relevant in *Brucella* infection. Previous data showed that the increase in glycolysis and lactate production favors *B. abortus* intracellular survival, suggesting that *Brucella* might take advantage of the metabolic change in macrophages to support bacterial growth (35). Accordingly, we demonstrated here that IRE1α participates in the metabolic shift during *B. abortus* infection, while previous reports indicated that the UPR favors *Brucella* replication in macrophages (9, 11). Thus, our results indicate that the IRE1α-induced metabolic shift may be involved in *B. abortus* survival. On the contrary, another report showed that during chronic infection, *Brucella* replicates preferentially in anti-inflammatory M2 macrophages which display a contrasting metabolic profile with decreased aerobic glycolysis. In this case, peroxisome proliferator-activated receptor γ (PPARγ)-mediated increase in glucose availability favors *Brucella* growth (43). Interestingly, M2 macrophages are more abundant during chronic brucellosis (13, 43). Hence, determining the influence of the UPR on macrophage metabolism during the course of *Brucella* infection may reveal exciting information about the role of UPR-induced metabolic changes in bacterial replication.

**Figure 8:**
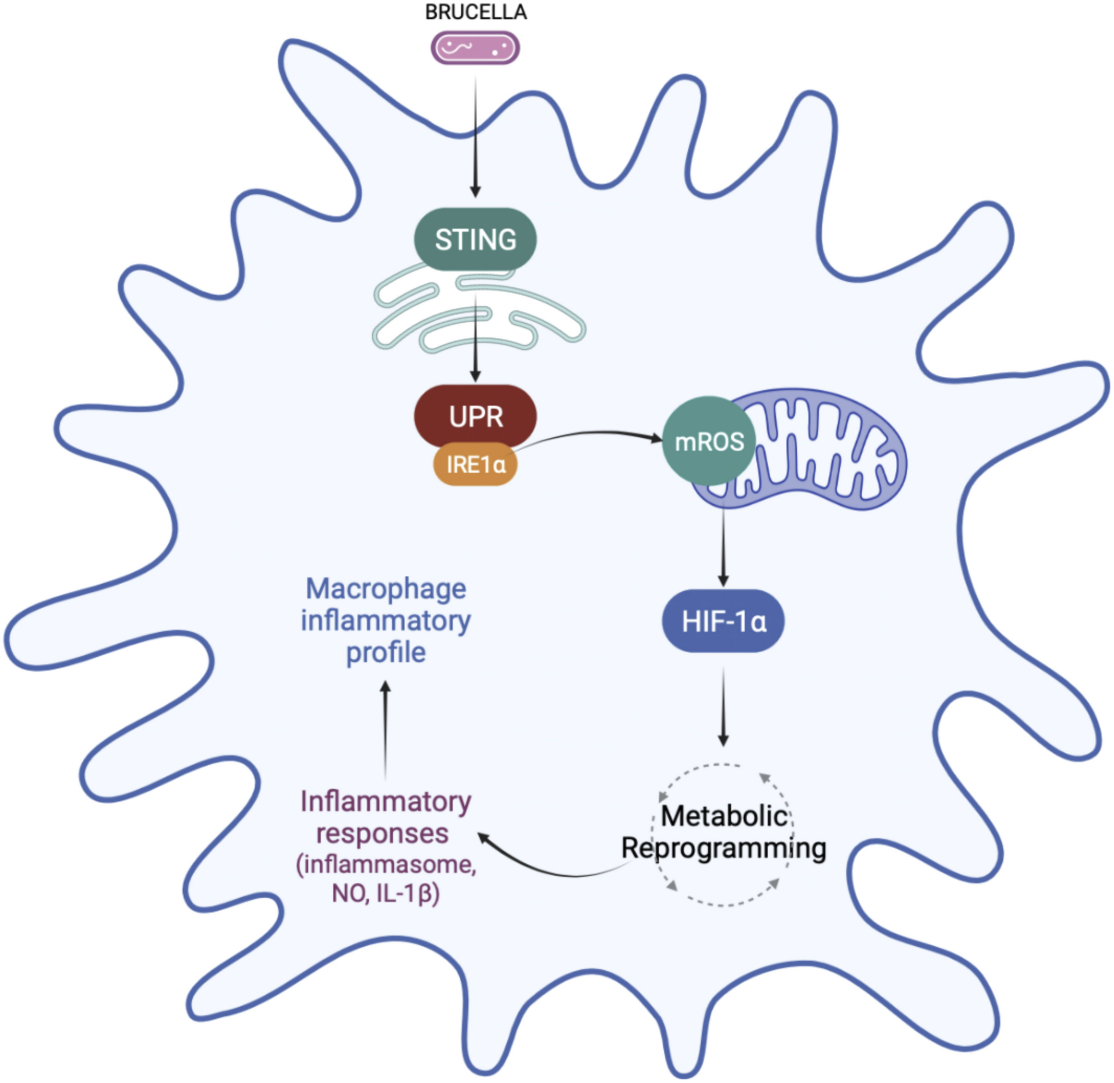
Regulation of macrophage metabolic function by IRE1α in *Brucella abortus* infection. IRE1α activation by *Brucella abortus* triggers mROS generation that induces HIF-1α stabilization in infected macrophages. IRE1α drives the metabolic reprogramming in macrophages, contributing to the enhanced glycolysis and reduced OXPHOS observed in *Brucella*-infected macrophages possibly *via* HIF-1α. This IRE1α-dependent HIF-1α stabilization is crucial for inducing inflammatory responses during infection. IRE1α induces inflammasome assembly, IL-1β release and NO production, supporting the inflammatory profile in macrophages infected with *B abortus*. Figure created using BioRender (https://biorender.com).

In summary, the data presented here reveals that IRE1α favors M1 inflammatory polarization and regulates macrophage metabolism. We demonstrated that IRE1α activation increases mROS production, which may contribute to the stabilization of HIF-1α, an important regulator of macrophage metabolic function during *B. abortus* infection. Accordingly, IRE1α contributes to the metabolic reprogramming of macrophages, favoring the glycolytic phenotype during infection. In addition, IRE1α favors inflammatory cytokine secretion, NO production and inflammasome activation (**Figure 8**). The results presented here revealed an important link between UPR and HIF-1α, which ultimately regulates macrophage metabolic profile and the immune response against *B. abortus* infection. Finally, our findings improve the understanding of how these UPR-induced immunometabolic changes impact bacterial pathogenesis.

## Data availability statement

The raw data supporting the conclusions of this article will be made available by the authors, without undue reservation.

## Ethics statement

All experiments involving animals were conducted in accordance with the Brazilian Federal Law number 11,794, which regulates the scientific use of animals in Brazil, the Institutional Animal Care and Use Committees (IACUC) guidelines, and the Animal Welfare Act and Regulations guidelines established by the American Veterinary Medical Association Panel on Euthanasia. Animals were fed, housed, and handled in strict agreement with these recommendations. All protocols were approved by the Committee for Ethics in Animal Experimentation (CEUA) at UFMG under permit #87/2017.

## Author contributions

EG, MG and SO devised the project and the main conceptual ideas. EG, MG, RS, KM, FM, designed and carried out the experiments. EG analyzed the data and prepared the figures. EG and SO wrote the manuscript with the input of all authors. SO provided the funding acquisition, supervised the project, reviewed, and submitted the manuscript. All authors contributed to the article and approved the submitted version.
